# Psychometrics of Persian version of the 11 items De Jong Gierveld loneliness scale among an Iranian older adults population

**DOI:** 10.1186/s12889-021-12068-x

**Published:** 2021-11-10

**Authors:** Lida Hosseini, Erika Sivarajan Froelicher, Hamid Sharif Nia, Mansoureh Ashghali Farahani

**Affiliations:** 1 Nursing Care Research Center, School of Nursing and Midwifery, Iran University of Medical Sciences, Tehran, Iran; 2 Department of Physiological Nursing, School of Nursing, University of California Sand Francisco, San Francisco, California USA; 3 Department of Epidemiology & Biostatistics, School of Medicine, University of California Sand Francisco, San Francisco, California, USA; 4 School of Nursing and Midwifery Amol, Mazandaran University of Medical Sciences, Sari, Iran

**Keywords:** Social loneliness, Emotional loneliness, Psychometrics, Validity, Reliability

## Abstract

**Background:**

Older adults duo to circumstances of aging such as relationship losses, medical morbidities, and functional declines, are prone to social isolation and loneliness more than any other age group. Furthermore, with The recent outbreak of the COVID-19 pandemic and the need to quarantine, the possibility of feelings of loneliness, especially in older adults, became an important nursing priority. Therefore, it is important to quickly identify loneliness and respond appropriately to prevent, reduce, or treat it. The aim of this study was to translate the De Jong Gierveld loneliness scale into Persian for older adults.

**Methods:**

The sample was 400 adults aged 65 and older with a mean age of 71.32 (SD= ± 6.09) years. Recruitment and data collection was done via online methods. The original scale was translated into Persian using the World Health Organization (WHO) protocol of forward-backward translation technique. Face validity and content validity; was followed by exploratory and confirmatory factor analysis. Lastly, reliability was assessed using the Average Inter-Item Correlation, Cronbach’s alpha, and McDonald’s Omega.

**Results:**

The results showed that the Persian version of the loneliness scale had two factors namely social loneliness (5 items) and emotional loneliness (3 items) and the combined score explained 45.66% of the total variance of this scale. In addition, all goodness of fit indices confirmed a two factors model fit and all of the reliability indices were excellent.

**Conclusions:**

The Persian version of the loneliness scale is useful and suitable for detecting social loneliness and emotional loneliness in older Iranian adults.

## Background

Older adults du to circumstances of aging such as relationship losses, medical morbidities, and functional declines, are prone to social isolation and loneliness more than any other age group [1]. The studies have shown, loneliness and social isolation are a major problem that has a negative effect on physical, mental health and longevity of older adults [2, 3]. The recent outbreak of Coronavirus in the 2019 (COVID-19) pandemic requiring quarantine has added to the problem of older adults becoming socially isolated. Since this disease is transmitted through respiratory droplets, the main way to control the spread of this virus in the population is social distancing, self-isolation, and quarantine [4]. Because older adults have weaker immune systems and chronic conditions such as heart disease, diabetes, lung disease, and cancer, they are most vulnerable in this pandemic, and greater emphasis is placed on their quarantine [2].

Social distancing, quarantine, and self-isolation can significantly change people’s daily lives and lead to immediate disruption of physical, social, and economic functions and can lead to mental health and psychological problems for persons, especially in older adults [5]. Based on a literature review, common psychological problems such as anxiety, depression, and increased loneliness were observed, especially in older adults after the quarantine [6–8].

A common definition for loneliness is” a state of solitude or being alone” or “inability to find meaning in one’s life” [9]. A “feeling of disconnectedness or isolation” is another way to define loneliness [10]. The prevalence of loneliness was reported in the general population in England as 5%, and in Germany as 11%, [11, 12]. It is noteworthy that the percentage of moderate and severe loneliness among older adults aged 65 and older is higher than in younger age groups. The prevalence of loneliness was 11% in older adults aged 65 in the United States; 40% in Australia, 24.1% in Israel [12]. The evidence shows that the loneliest age groups are those above 75 years [12]. Loneliness was also reported among older adults in an Iranian sample. Specifically, 24% were moderately and 5% were feeling severely lonely [13]. Loneliness is an important problem because even before the coronavirus crisis, loneliness was a public health problem and a major concern in older adults. Because studies have shown that 35% of adults 45 years and older and 43% of adults over 60 years experience loneliness at least “sometimes” [14].

Studies have shown that loneliness is associated with numerous physical and psychological problems in older adults. The presence of loneliness increased the risk of heart disease by 9%, the risk of stroke by 32%, and the risk of dementia by 50% [6, 15]. Also, loneliness was associated with low self-esteem, fatigue, depression [16], lack of goals, unpleasant thoughts, negative thoughts, suicidal thoughts, thoughts of death [17], social disorders, loss of social interaction, sleep and anxiety disorders, frustration, mobility limitation [18, 19] and increased mortality [19]. Also, a strong association has been observed between loneliness and depression [20].

Given the importance of loneliness, Weis (1973) distinguished between two dimensions of loneliness; namely emotional loneliness and social loneliness [21]. Social loneliness means the “mental assessment of a situation in which the person finds the level of relationships with their friends and colleagues less than desirable”. Emotional loneliness refers to the “lack of existence of relatives or others on whom one is emotionally dependent”. Emotional loneliness is more about family status [22]. Weis proposed that the effects of either of these two loneliness domains could be different. For example, he stated that emotional loneliness leads to feelings of aloneness, anxiety, over-monitoring, hypersensitivity to minimal symptoms, and feelings of abandonment. In contrast, social loneliness is associated with boredom, depression and aimlessness. Also the type of loneliness varies from person to person depending on their demographic characteristics and their conditions [23]. In general, it has been shown that in both types of loneliness, the existence of a specific severity of deprivation was considered as the essence of loneliness [19].

Globally, older people need health care more often than younger populations. Nurses have the most contact with this population; Therefore, nurses are ideally positioned to identify older adults at risk of loneliness and its severity. They can create appropriate clinical and public health interventions to prevent, reduce or treat loneliness. Nurses can also screen patients using a reliable and valid scale to assess loneliness accurately. One of the most important scales available to measure loneliness is the De Jong Gierveld Loneliness Scale, developed in 1985 [24]. This scale consists of 11 items that examine the feelings of loneliness and distinguishes between social and emotional loneliness. Of the 11 items, six items measure emotional loneliness with negative semantic load, and five items identify social loneliness with sentences using positive semantic load. The validity and reliability of this scale have been evaluated in several European and Asian countries such as Brazil [25], Chinese [26], Netherland [27], Poland [28], and Spanish [29], but not in Iran. All of these studies confirmed that this scale is suitable, reliable, and valid for the assessment of loneliness among older adults. Due to the increase in the aging population, as well as inappropriate attitudes toward older adults, the rate of social isolation and loneliness is high among older Iranian adults [30]. Additionally, the necessity for quarantining has made older adults more prone to loneliness during the COVID-19 pandemic [31, 32]. Therefore, there is a heightened need to accurately identify and distinguish between the two types of loneliness. Because there is an important difference between feeling lonely “due to losing someone” and feeling lonely “due to the lack of a social network”, their side effects and interventions differ depending on the type of loneliness [33]. The aim of this study was to translate and evaluate the psychometric properties of the “11 items De Jong Gierveld Loneliness Scale” and to test the feasibility of a Persian version of among older adult population. The reasons for choosing this scale where it’s simplicity, brevity, practicality, comprehensiveness, and to be able to distinguishing the two different types of dimensions of loneliness.

## Methods

### Study design

This study used a cross-sectional design.

### Sample

We recruited a sample of older adults in Tehran between May 2020 until August 2020. The inclusion criteria were: adults 65 years of age and older, who had the ability to use a social network, gave written consent to participate in the study, were Iranian, were fluent in Persian, and are free of cognitive and memory deficits. The sample size was determined based on the number of items in the scale multiplied by 10 (11 × 10 = 110) [34]. To ensure a sufficient sample size, we included 400 (200 for the EFA stage and 200 for the CFA stage) older adults in the study. We created the online questionnaire via Google Forms and sent its URL link by email or social networking applications such as a Telegram channel or WhatsApp to the target population. After that, data were extracted from Google Form in the Excel file and prepared for analysis. Samples were selected by a non-random and snowball method through social groups related to the older adults and introducing people. The consent form was also obtained from participants at the beginning of the questionnaire.

### Measurements

We used two questionnaires: a demographic questionnaire and the Persian version of De Jong Gierveld Loneliness Scale in this study. The demographic questionnaire consisted of personal information including age (65 years and older), gender (female, male), marital status (single, married, divorced, and widow), level of education (illiterate, less than a diploma, diploma, and academic), economic status (low, medium, and high), employment status (unemployed, employed, retired, housewife, and free work), and the number of children (none, one, two, three, and four and more). The original Loneliness Scale assessed the status of loneliness. This 11-item scale including two factors: social loneliness (5 items) with positive semantic load for assessing feelings of belongingness, and emotional loneliness (6 items) with negative semantic load for assessing feelings of social loss or disappointment. This scale is a valid and reliable scale for assessing emotional, social, and overall loneliness. The Loneliness scale uses response options on a 5-point Likert-type scale, where 0 = None of the time, 1 = Rarely, 2 = some of the time, 3 = often, and 4 = All of the time [9]. Response categories were “no”, “more or less”, and “yes”. Positive answers (“more or less”, “yes”, or “yes!”) on items 2, 3, 5, 6, 9, 10 provide an emotional loneliness score and negative answers (“no!”, “no”, or “more or less”) on items 1, 4, 7, 8, 11 provide the social loneliness score. After reversing the positive items of the scores on the loneliness scale range from 0 (not lonely) to 11 (extremely lonely) with a reliability value of .81 [35].

### Translation

The process of translation and back translation used in the development of the Persian version of this scale was based on the WHO (2014) protocol of forward-backward translation technique [36]. First, we obtained the written permission from Professor De Jong Gierveld, the developer of the scale via e-mail to be able to translate and validate the questionnaire. Second, we asked two English-Persian translators to translate the Loneliness Scale independently. Third, two Persian translations of the questionnaire were reviewed and evaluated by an expert panel (including some of this paper’s authors (H.SH and M.F) as well as two professional translators) and after reviewing both translations and discussing the differences between them, we created the Persian version of this questionnaire. Fourth, two English-Persian translators (unlike the first two translators) who had no knowledge of the English version of the questionnaire, back-translated the Persian version into English. Lastly, an expert panel reviewed the two English back-translations. After the necessary revisions and adjustments, the final English version was sent to De Jong Gierveld for confirmation by email.

### Face validity

Face validity was established using both qualitative and quantitative methods. For the qualitative step, we gave the scale to 10 older adults aged over 65 years and asked them to comment on the appropriateness of the appearance, degree of clarity or ambiguity of the selected words, and the rationale for the sequence of the items. These viewpoints were included in the final version. Then the final version was assessed using quantitative face validity by measuring item impact scores. To perform this phase of validity, 10 target population members were asked to rate items on a five-point scale; where 5 = quite important, 4 = somewhat important, 3 = medium important, 2 = slightly important, and 1 = not at all important. An impact score greater than 1.5 was considered appropriate. The impact score was calculated using the following formula: (Impact score = frequency (%) importance) [37].

### Content validity

Both qualitative and quantitative methods were used to calculate content validity. To determine the quality of the content validity, indicators such as grammar, use of appropriate words, and item allocation were evaluated by reviewing the opinions of 10 experts in the fields of measurement, psychology, and aging. In addition, to assess the content validity quantitatively, we measured content validity ratios (CVR) and content validity index (CVI) via modified kappa coefficient (K). To calculate CVR, the questionnaire was administered to 10 persons representing education, psychometrics, psychology, and aging were asked to evaluate how essential each item was on a three-point scale as follows: 1 = Not essential, 2 = Useful but not essential, and 3 = Essential [38, 39]. Then the CVR was evaluated using the following formula: CVR = (ne – [N/2])/(N/2). In this formula, nE is the number of experts who consider an item “essential” and N is the total number of experts on the panel. Since the number of the expert panel was 10, based on Criterion in the Lawshe table, the minimum acceptable CVR is equal to 0.62 [40]. Also items relevancy of the 11-items scale was evaluated by 10 experts on a four-point scale as follows: 1 = irrelevant, 2 = somewhat relevant, 3 = quite relevant, 4 = highly relevant. For evaluation CVI, the modified kappa coefficient (K), which is an important complement to CVI, was calculated to determine the degree of chance agreement of experts and to eliminate the risk of chance effect for each item was evaluated using the following formula: K = (I-CVI – Pc) / (1 – Pc). Evaluation criteria for Kappa are as follows: good = 0.60–0.74 and the excellent value of Kappa > 0.75 [41].

### Construct validity

Construct validity was evaluated using Exploratory Factor Analysis (EFA), Confirmatory Factor Analysis (CFA) and convergent and divergent Validity. Based on this criterion for factor analysis, 10 subjects for each item of scale were needed. Thus, a sample of 400 older adults was considered sufficient for two stages of EFA and CFA (each stage 200) [42].

### Exploratory factor analysis (EFA)

The first sample of 200 was randomly selected from the total samples for EFA. Then EFA was performed with Maximum Likelihood Exploratory Factor Analysis (MLEFA) with Promax rotation. The quality of the response and the quality of the samples was calculated with Kaiser-Meyer-Olkin (KMO) and Bartlett test, where acceptable values for the KMO index are greater than 0.7. Furthermore, 95% confidence intervals (CIs) were estimated for each eigenvalue based on CI 95 width (z: 1.96). Also, Horn’s parallel analysis approach was used to determine the number of latent factors that items with communalities < 0.2 were excluded from EFA [43]. The number of extracted factors was determined based on three modern approaches: a) Exploratory Graph Analysis (EGA), b) parallel analysis, and c) parallel analysis scree plot [44]. Items with factor loading values of 0.3 or greater were considered appropriate. Based on the three-indicator rules, at least three items must exist for each factor and the presence of a single item in the factor was estimated approximately 0.3 based on the formula CV = 5·152 ÷ √(*n* − 2), (in this formula, the ‘CV’ is the number of extractable factors and ‘n’ is the sample size) [45].

### Confirmatory factor analysis (CFA)

For this step, the structure obtained through EFA was investigated by CFA. The most important objective of CFA is to determine the power of a predefined factor model, which in the present study was the same structure as obtained by EFA, with a set of observed data [46]. In CFA, the model fitness was assessed according to the Parsimonious Normed Fit Index (PNFI), Parsimonious Comparative Fit Index (PCFI) and Adjusted Goodness of Fit Index (AGFI) (> 0·5), Comparative Fit Index (CFI), and Incremental Fit Index (IFI) (> 0·9), Root Mean Square Error of Approximation (RMSEA) (> 0·08), and Minimum Discrepancy Function divided by Degrees of Freedom (CMIN/DF) (< 3) [47].

### Convergent and divergent validity assessment

CFA is a multimethod-multi-trait approach suitable for construct validity that covers convergent and divergent validity. To determine convergent and divergent validity, the correlation between variables was determined using AMOS24 software, and then the weighted standardized regression table was determined. Finally, using Gaskin’s coded Excel software, convergent and divergent validity was obtained [48]. Convergent and divergent construct validity of the concept of loneliness was measured by the Fornell and Larker approach based on the following parameters: The Average Variance Extracted (AVE) and Maximum Shared Squared Variance (MSV). For convergent validity, the AVE should be greater than 0.5, and for the divergent validity, the MSV must be less than AVE [49].

### Reliability assessment

Reliability is the stability and repeatability of a tool. In this study, internal consistency was estimated using Cronbach’s alpha (α), McDonald’s omega (Ω), and Average inter-item Correlation (AIC). Coefficients Ω and α values greater than 0.7 were acceptable [50]. The AIC value between 0.2 and 0.4 indicated good internal consistency [42]. The composite reliability (CR), which replaces Cronbach’s alpha coefficient in structural equation modeling were evaluated. The CR values greater than 0.7 were considered acceptable [51].

### Multivariate normality and outliers

Univariate distributions were examined for outliers, skewness, and kurtosis. Multivariate distributions were evaluated for normality and multivariate outliers. Multivariate normality was assessed with Mardia’s coefficient of multivariate kurtosis. One indication of deviation from a normal distribution is a Mardia’s coefficient greater than 8 [52]. Multivariate outliers were evaluated through the evaluation of a Mahalanobis distance. Items with a Mahalanobis distance of *p* < .001 were considered to be multivariate outliers [52]. All of the statistical analyses were performed by SPSS_26_, SPSS-R menu_2_, AMOS_24,_ and JASP_0.14.0.0_ software.

### Ethics statement

Ethical approval for this study was obtained from the Ethics Committee of Mazandaran University of Medical Sciences (Code: IR.MAZUMS.REC.1399.6682), Sari, Iran.

## Result

### Demographic characteristics

In this sample (*n* = 400) the mean age and standard deviation (SD) was 71.32 (SD)= ±6.09) years. The majority of the sample were men (60.5%), retired (43%), married (82.8%), with a high level of education (55.3%) and “high” financial level (56.8%). Details of the demographic characteristics of the sample are shown in Table 1.

**Table 1:** Demographic characteristics of participants (*n* = 400)

Variables	% (n)
Gender	
Female	39.5 (158)
Male	60.5 (242)
Marital status	
Single	7.3 (29)
Married	82.8 (331)
Divorced	3.3 (13)
Widowed	6.8 (27)
Education level	
Illiterate	4.5 (18)
Less than diploma	20.3 (81)
Diploma	20.0 (80)
BS/ MS/ PhD	55.3 (221)
Employment	
Unemployed	6.0 (24)
Employed	26.3 (105)
Retired	43.0 (172)
Housewife	14.0 (56)
Volunteer	10.8 (43)
Economic status	
Low	11.0 (44)
Medium	32.3 (129)
High	56.8 (227)
Number of children	
None	11.0 (44)
One	10.5 (42)
Two	33.0 (132)
Three	22.5 (90)
Four and more	23.0 (92)

### Face and content validity

Based on the result of the face validity, all items of the scale were appropriate, clear, and relevant and the impact score was greater than 1.5. In addition, the content validity using CVR of all items was appropriate according to the Lawshe table and the kappa coefficient (K) of all items was higher than 0.75.

### Construct validity

**Fig. 1:**
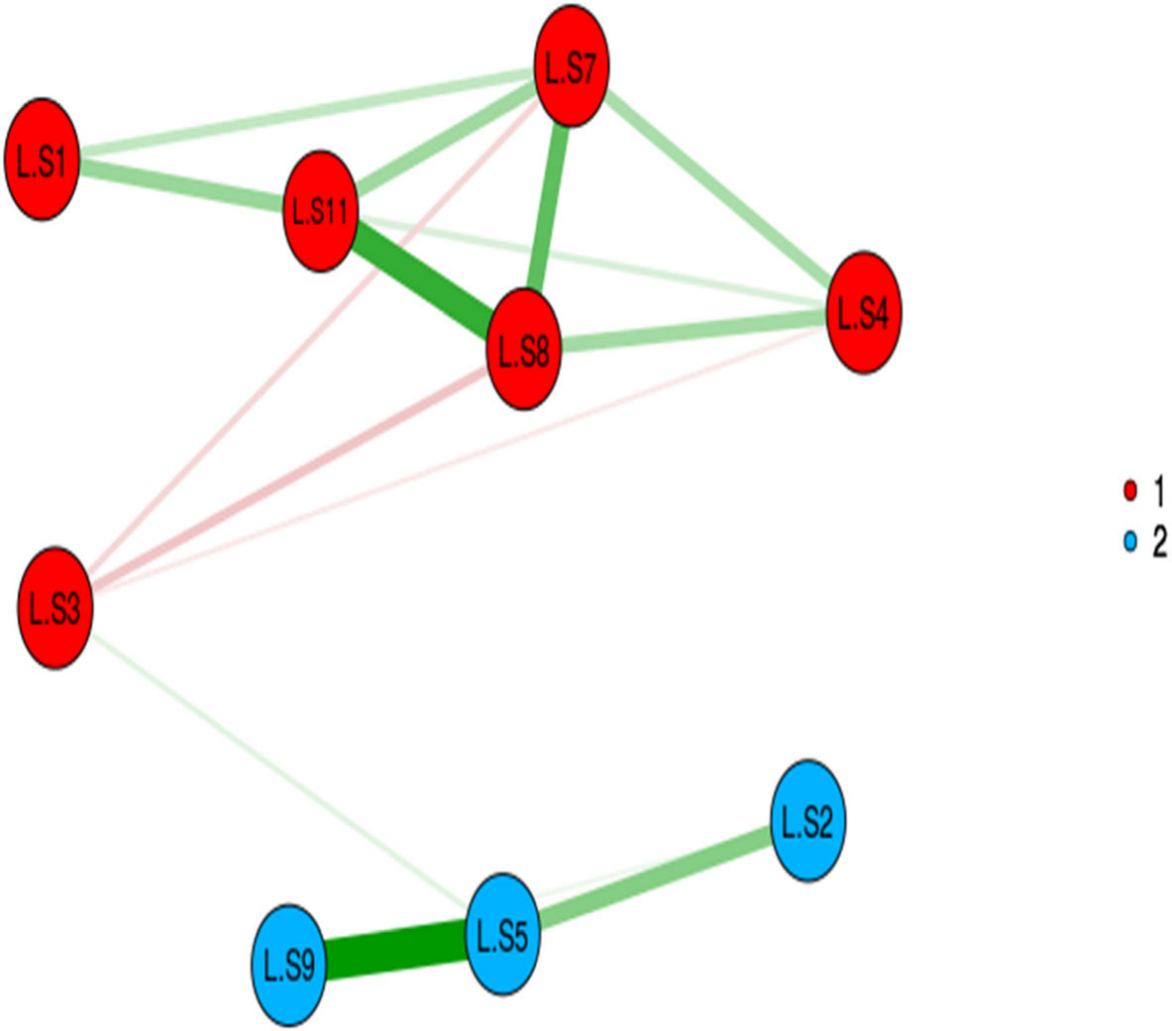
Exploratory Graph Analysis

**Fig. 2:**
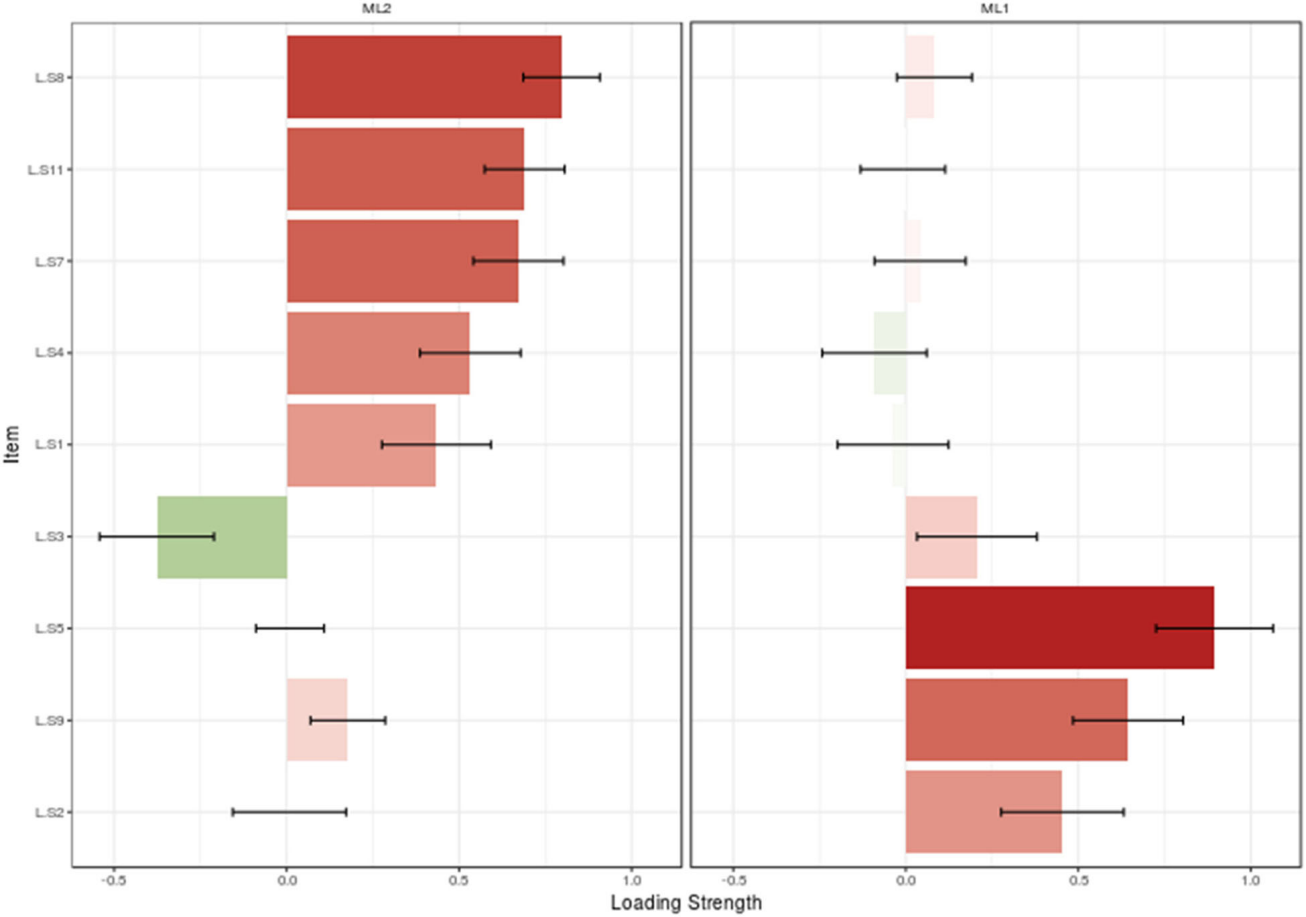
Loading strength of items in factors

**Fig. 3:**
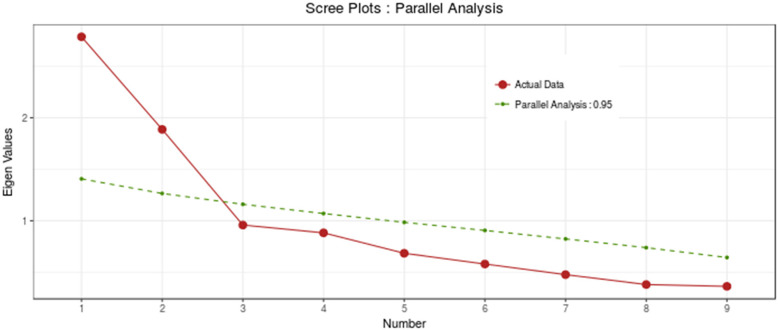
The parallel analysis scree plot

The results of MLEFA showed that the KMO test value was 0.817 and Bartlett’s test value was 1018.90 (*P* < 0.001). It also revealed two factors extracted in the MLEFA approach for Loneliness Scale. See details in Figs. 1, 2, and 3. These two factors (factor one with items of 1,4,7,8,11 and factor two with items of 2,5,9) identified in the EFA of the present study confirmed the factors of the original loneliness scale with an explained total variance of 45.66% in this sample. Three items (items of 3, 6, and 10) of the original scale were extracted in EFA because their factor loading was lower than 0.3. The Eigenvalues and percent of variances of these two factors are shown in Table 2.

**Table 2:** Exploratory factors extracted of De Jong Gierveld Loneliness Scale (*n* = 200)

Factors	Q_n_. Item	Factor loading	h^2^	Eigenvalue	%Variance
Social loneliness	8. There are enough people I feel close to.	0.846	0.699	2.77	30.84
	7. There are many people I can trust completely.	0.741	0.540		
	4. There are plenty of people I can lean on when I have problems.	0.701	0.502		
	11. I can call on my friends whenever I need them.	0.669	0.447		
	1. There is always someone I can talk to about my day-to-day problems.	0.538	0.293		
Emotional loneliness	5. I miss the pleasure of the company of others.	0.799	0.637	1.33	14.81
	9. I miss having people around me	0.657	0.416		
	2. I miss having a really close friend.	0.571	0.371		

* h^2^: Communalities

Note: Factor loading for three items 3 (0.287), 6 (0.222), and 10 (0.010) were lower than 0.3 and thus they were removed

**Fig. 4:**
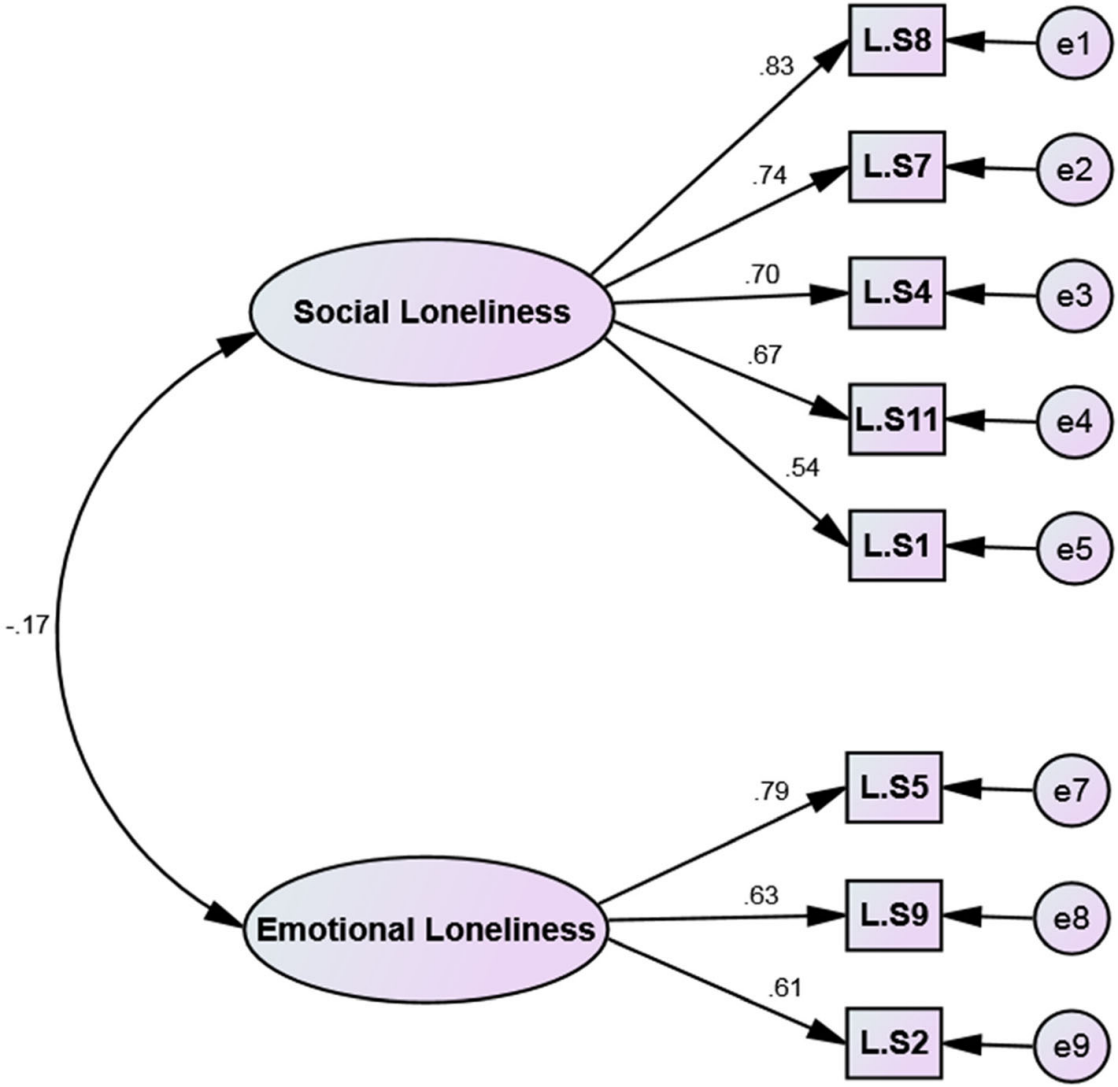
First order CFA of De Jong Gierveld Loneliness Scale (*n* = 200)

Based on the results of the CFA, the extracted model after it was confirmed by all goodness of fit indices. Details of these indices are shown in Table 3 and Fig. 4. The reliability of the two factors of this scale was excellent based on the Cronbach’s alpha, McDonald’s Omega, CR. Details of these indices are shown in Table 4. As well as the AIC values of factors were good. Based on results of convergent validity, the AVE of two factors was more than the MSV and shows that the factors have good convergent, but no discriminant validity (Table 4).

**Table 3:** Fit indices of the CFA Model after Structure Modification of the Persian Loneliness Scale (*n* = 200)

Indices	χ^**2**^	df	***P*** value	CMIN/DF	RMSEA (CI90%)	PNFI	PCFI	TLI	IFI	CFI
CFA Model after Structure Modification	28.570	19	< .0001	1.50	.036 (.030 to .061)	.658	.671	.985	.990	.990

*DF* Degree of freedom, *PCFI* Parsimonious Comparative Fit Index, *PNFI* Parsimonious Normed Fit Index, *CMIN/DF* Minimum Discrepancy Function divided by Degrees of Freedom, *RMSEA* Root Mean Square Error of Approximation, *TLI* Tuker-Lewis Index and *CFI* Comparative Fit Index, *IFI* Incremental Fit Index

Fitness indexes: PNFI, PCFI (> 0.5); TLI, IFI, CFI (> 0.9), RMSEA (<0.08), CMIN/DF (<3 good, <5 acceptable)

**Table 4:** The indices of the convergent, discriminant validity, and internal consistency of Loneliness Scale (*n* = 400)

	CR	AVE	MSV	Alpha [CI95%]	Omega	AIC
**Social loneliness**	0.851	0.493	−0.17	.822(.79 to .84)	.828	.484
**Emotional loneliness**	0.763	0.464	−0.17	.711(.65 to .75)	.719	.454

DF: *CR* composite reliability, *AVE* Average Variance Extracted, *MSV* Maximum Shared Squared Variance, *AIC* Average inter-item Correlation

## Discussion

In this study, we assessed the psychometric properties of the Persian version of the Loneliness Scale among an Iranian older adults population during the COVID-19 pandemic. Items measuring the social and emotional loneliness factor explain more than 46% of the variance of the concept of loneliness. Since the two factors were eventually extracted, these two factors did not have discriminant validity. Furthermore, based on our results, the Persian version of the Loneliness Scale showed a clear factor structure with two factors, namely social loneliness (5 items) and emotional loneliness (3 items). All reliability indices such as Cronbach’s alpha, McDonald’s Omega, CR, and maximal reliability were excellent for these two subscales.

The psychometric properties of this scale performed well in several countries. Most of them also confirmed the two dimensions: social and emotional loneliness like the original scale. For example, Grace Tak Yu Leung et al. validated the Chinese version containing 6-items of the De Jong Gierveld Loneliness Scale (short version) among 103 Hong Kong Chinese community-dwelling older adults [26]. They confirmed the two dimensions of loneliness (social and emotional) and Cronbach’s α of the 6-item scale was 0.76 in their sample. Another study was performed by Pawel Grygiel et al. (2013) year in the Polish version of this scale and they assess the psychometric features of their scale [53]. They tested this scale through analysis of differential item functioning (DIF) and confirmed two dimensions of loneliness (social and emotional) which generalize into a higher-order factor of a general sense of loneliness (bifactor structure). The reliability of this scale was (α = .89) and homogeneity was (H = .47). Esther Iecovich (2013) year also evaluated for validity and reliability of the Hebrew version of the De Jong Gierveld Loneliness Scale among 2100 older adults in three sub-samples in Israel [54]. Based on exploratory factor analysis, they extracted three dimensions instead of two-dimension including emotional loneliness 1 (items of 1, 2, 3), social loneliness (items of 4, 5, 6, 9, 11), and emotional loneliness 2 (items of 7, 8, 10). In fact, the items’ number of social and emotional dimensions were similar to the original scale. The emotional dimension is split into two separate dimensions. The items assigned to each dimension are different from the original scale, while in our study the items assigned to each dimension were completely consistent with the original scale. Jose *M. toma*’s et al. (2017) designed a study to assess the validity and reliability of the Spanish version of the De Jong Gierveld Loneliness Scale among 335 people aged 55 years or older [29]. Their results confirmed a unidimensional substantive structure, but with minor method effects associated with negatively worded items such as items 3, 9, and 10. They concluded that the multidimensional IRT analysis, the 2 Parameters Logistic Model provides better information functions than the Rasch model. As well as reliability (composite reliability index (CRI) was .89) and validity of the Spanish version of this scale estimates were adequate. Similar to our finding, this study showed that items 3 and 10 have very high difficulties and Jose *M. toma*’s stated that people feel deeply lonely and may agree with these items. In our study these item had factor loading less than 0.3 and were therefore removed. Therefore, we conclude that our participants may not have felt extremely lonely on the Persian loneliness scale. Patrícia Nunes da Fonseca et al. (2018) also assessed the validity and reliability of short version of De Jong Gierveld Loneliness Scale in the Brazilian context in the general population [25]. Their finding confirmed two dimensions of loneliness (social and emotional) and showed that it is psychometrically suitable for use in Brazil.

Based on the review of studies conducted in the evaluation of psychometric properties of the loneliness scale in the older population, it can be concluded that the findings of the present study are consistent with previous findings. The findings of our study confirmed two factors such as social and emotional loneliness with the same items of the original scale.

The first factor of this scale is ‘social loneliness’ with 5 items refers to a lack of social networks. It is noteworthy that items of social loneliness factor confirmed in confirmatory factor analysis, are identical to the original scale items in this sample. According to Weis (1973), social loneliness means the lack of a network of social relationships in which a person is part of a group of friends who share common interests such as their feelings, ideas, dreams, and activities [21]. One study has shown that older adults who have retired from their professional lives may experience more social loneliness [55]. Moreover, during the COVID-19 pandemic, the WHO has advised maintaining social distance, quarantine, and self-isolation to reduce the prevalence of this disease in the population, especially in vulnerable groups including older adults [4]. This has resulted in a reduction of social relationships, especially in older adults. This in turn can make them more vulnerable. This amplifies the chance of developing several negative outcomes such as depression, distress, anxiety, decreasing life satisfaction among this population during quarantine for the COVID-19 pandemic [18, 19]. Therefore, it is important to pay attention to loneliness.

The second factor of this scale is ‘emotional loneliness’ consisting of three items that refer to a lack of close and intimate attachment to another person [55]. According to Weis (1973), persons who experience emotional loneliness miss other individuals. This often occurs after losing a close emotional attachment through a divorce or the death of a partner [56]. Recent studies have shown that emotional loneliness is more harmful to health than social loneliness, and this can cause problems such as feelings of aloneness, anxiety, hypervigilance, high sensitivity to minimal cues, and feelings of abandonment [23]. In the study by Súilleabháin and Steptoe emotional loneliness in older adults living alone is significantly associated with an increased risk of mortality [23]. It is noteworthy that out of the total items of this subscale of the original scale, three items refer to missing someone. Given the quarantine of these individuals in this pandemic situation, our finding during EFA and CFA extracted these three items for this subscale. In fact, it shows the impact it has on these people in losing their emotional relationships with their relatives.

### Limitation

The most important limitations of this study resulted from online data collection during the COVID-19 pandemic. Therefore, people who did not have access to the internet or social networks were not included in our sample. An older adult who was technically less astute may likely have been underrepresented in this sample. This sample may therefore not be representative of all older adults. Furthermore, since our sample was not randomly selected, bias may have occurred. For this reason, the findings need to be interpreted with caution.

## Conclusion

Based on this study, we conclude that the Persian version of the loneliness scale can detect two distinct dimensions of loneliness; such as social loneliness and emotional loneliness in older adults with 8 items. Items three, six, and 10 on the original scale are about feelings of loss, emptiness, and rejection that they may reflect feeling deeply lonely. These items were deleted in our scale, given the prevailing conditions in society and quarantine, and as the results show, these people seem to feel lonelier because of limited access to their existing relationships, not due to a lacking relationship. Furthermore, this was especially true during quarantine. Nurses need a suitable scale to identify those who suffer from the negative health impacts of loneliness and need target interventions to improve their social conditions. This study provides a suitable scale for researchers and health care practitioners. Interventions are needed to provide relief from loneliness in older adults during the COVID-19 pandemic.

## Data Availability

The datasets generated and analyzed during the current study are available from the corresponding author on reasonable request.

## Abbreviations

CVR: content validity ratios
CVI: content validity index
EFA: Exploratory Factor Analysis
CFA: Confirmatory Factor Analysis
MLEFA: Maximum Likelihood Exploratory Factor Analysis
KMO: Kaiser-Meyer-Olkin
CIs: confidence intervals
EGA: Exploratory Graph Analysis
PNFI: Parsimonious Normed Fit Index
PCFI: Parsimonious Comparative Fit Index
AGFI: Adjusted Goodness of Fit Index
CFI: Comparative of Fit Index
IFI: Incremental Fit Index
RMSEA: Root Mean Square Error of Approximation
CMIN/DF: Minimum Discrepancy Function divided by Degrees of Freedom
AVE: Average Variance Extracted
MSV: Maximum Shared Squared Variance
AIC: Average inter-item Correlation
CR: composite reliability
